# Organic materials in the wall paintings in Pompei: a case study of *Insula* del Centenario

**DOI:** 10.1186/1752-153X-6-107

**Published:** 2012-09-24

**Authors:** Antonella Casoli, Sara Santoro

**Affiliations:** 1 Dipartimento di Chimica, Università degli Studi di Parma, V.le G. P. Usberti 17/A, 43121, Parma, Italy; 2 Dipartimento di Studi Classici dall'Antico al Contemporaneo, Università G.d'Annunzio, Chieti Pescara, Via dei Vestini 31, 66013, Chieti, Italy

**Keywords:** Pompei, Wall paintings, Paint technique, Organic material, Gas chromatography/mass spectrometry

## Abstract

**Background:**

The present research concerns the Roman wall paintings preserved at *Insula* del Centenario (IX, 8), the important Pompeian block situated in the Regio IX, along Via di Nola.

**Results:**

The aims of this research are two: to verify the presence of lipidic and proteinaceous material to spread the pigments, and to identify organic matter in painting materials owing to previous restoration works. The samples collected from the wall paintings of different rooms have been investigated by Fourier Transform Infrared Spectroscopy (FT-IR), and Gas Chromatography/ Mass Spectrometry (GC/MS).

**Conclusions:**

The analytical results show that these Roman wall paintings were realized without the use of lipidic and proteinaceous materials, supposedly in fresco technique. Moreover, it was detected that wax, egg, and animal glue were used in previous restoration works for protective purpose and to restore the wall paintings to their original brilliant colours.

## Background

The *Insula* del Centenario reemerged in 1879 (the 18^th^ Centenary of the Vesuvio’s eruption) and in 1880, but a fifth of its surface still remains unexplored. The area excavated is largely taken up by the Casa del Centenario, a building of great interest from an architectonic point of view because of its complexity (it has two entrance halls, one *peristyle*, one *nymphaeum* with mosaic fountain, a *criptoportico*, and one of the few, private thermal installations in Pompei), and also from a decorative point of view because of its mosaics and, above all, for its wall paintings (style I, initial style III, style IV). The rest of the block is representative of the average urban fabric of late Pompei; a house with a *peristyle*, a few modest houses, and a variety of establishments, *tabernae*, a *schola epicurea,* a *hospitium.*

In terms of dimensions (2.500 sq. metres), of quality of decorations and variety of structures, *Insula* del Centenario is one of the most important Pompeian complexes. The study, retrieval and increased value have elicited great interest from the scientific community, in spite of the fact that its importance has not been advertised and has remained closed to the public.

The paintings examined, of the IV Pompeian style, have strong colours and varied subjects (mythological, erotic, exotic, architectonic, ornamental) are all located *in situ.*

The study was realised as part of the Pompei Project “*Insula* del Centenario”, and agreed upon during a convention that took place in 1999, held by the University of Bologna in conjunction with the Soprintendenza Archeologica di Pompei, and inserted into the larger archeometric project aimed at identifying building materials, mortar, plaster, pigments, and mosaic work of the *Insula*[1]*.*

The present paper aims to know the painting techniques used in the wall paintings of *Insula* del Centenario in Pompei. This objective was conceived to add to what is already known on the techniques of Roman painting, on which recent archaeometric studies are working on and gradually making clearer [2-6]. We cite as an example works by Varone and Bearat on an excavation in Pompei along via dell’Abbondanza [7]. Here one can see how various painters may possibly have worked together, some in fresco and some in tempera. The authors indicate that some collected fragments could contain an organic substance, though there is no certainty about which type of substance.

The present research is based on the question as to whether the wall paintings of *Insula* del Centenario were painted in fresco or tempera. To achieve a wider range of documentation the wall paintings of the nearby Casa del Maiale have been taken into consideration. It was necessary to understand if different painting techniques were used in structures that had a different assignment or degree of importance.

Having found the answer to our first question (all the wall paintings considered were made a fresco), we turned to a second objective: that of individuating the organic substances applied to the pictorial surfaces during previous restoration interventions, and to determine at the same time which areas were restored by means of organic substances. This second phase of our research aims at assembling the totality of the restorations carried out on those paintings, for investigative or protective purposes in view of future restoration works.

We made use of Fourier Transform Infrared Spectroscopy (FT-IR) and gas chromatography combined with mass spectrometry (GC/MS). GC/MS is currently the most commonly used technique for binding media identification. This technique in particular permits the characterization of the organic components by the determination of the content of amino acids and fatty acids originating from proteins and fats respectively [8-10].

## Experimental

### Sampling

Small amounts of sample (0,1÷0.8 mg) were taken by scraping with a bistoury the surfaces of the paintings giving signs of damage. The samples obtained were carefully handled to prevent contamination. Efforts were also made to reduce to the minimum the number of samples collected for analysis.

**Figure 1:**
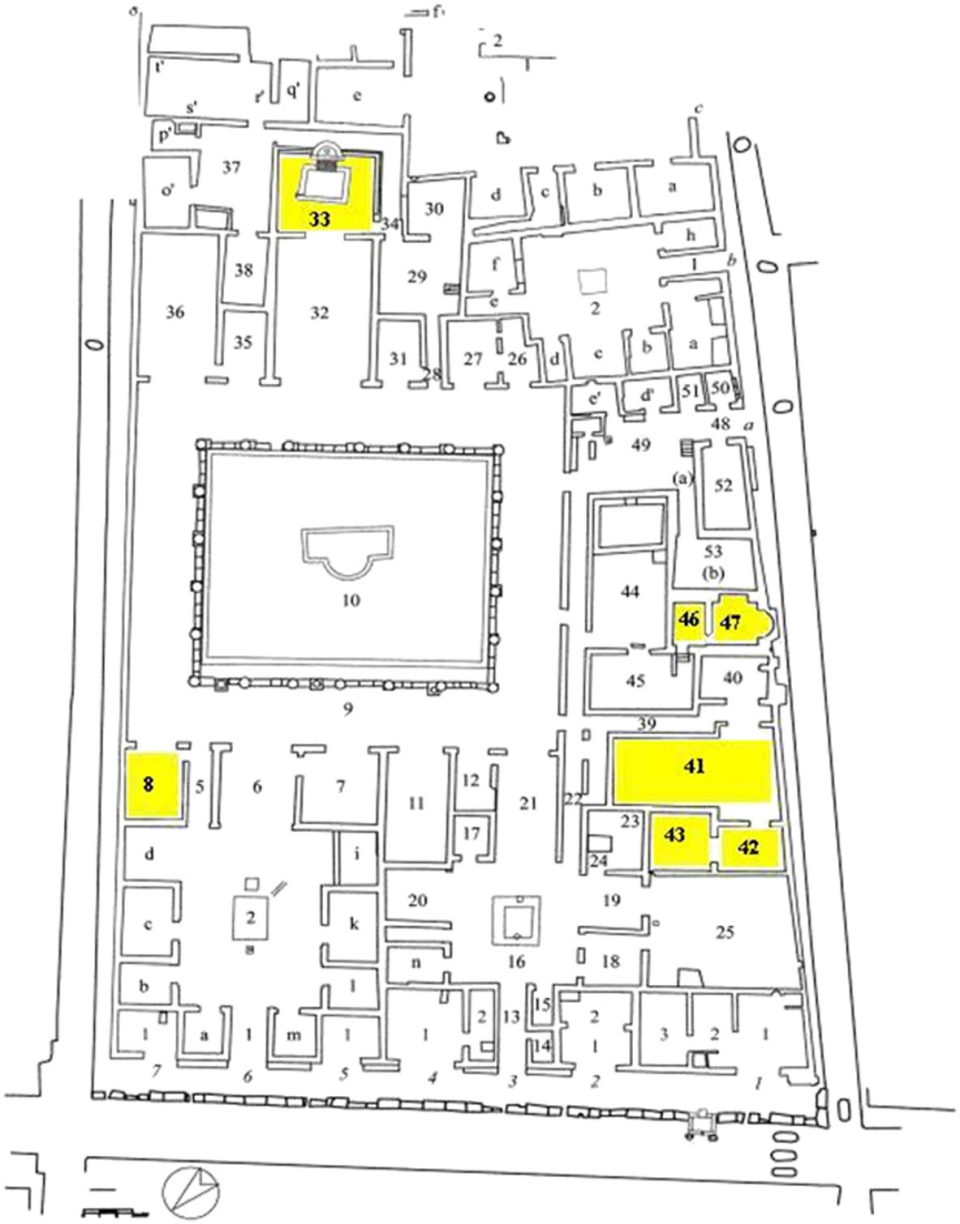
**Pompei, Map of *Insula *del Centenario (IX 8). **The rooms in yellow are those considered during the second sampling (June 2004).

In this research we have concentrated on the following rooms of the domus (Figure 1): 

– room 2 of the *Hospitium Hygine Firmi;*

– room 8, named black *oecus* on the basis of how its function was interpreted and because the colour black predominated in its decorations;

– room 9, large peristyle with twenty two columns, an important area representative of a typical *domus;*

– room 33, the *nymphaeum* with pictures depicting ferocious animals and a fountain richly decorated with mosaics;

– rooms 41, 42, 43 of the private apartment that includes a *triclinium* (dining room with triclinic beds)**,** the antechamber of the so-called erotic *cubiculum* and the *cubiculum*, that had the paintings of erotic images;

– rooms 46 and 47, *tepidarium* and *calidarium* of the private thermal installation of the *domus;*

– room 2, corresponding to the *atrium* of the *Hospitium Hygini Firmi;*

– two rooms, 6 and 11, of the adjacent Casa del Maiale (IX 9, c), to operate a comparison with the paintings of another building.

**Figure 2:**
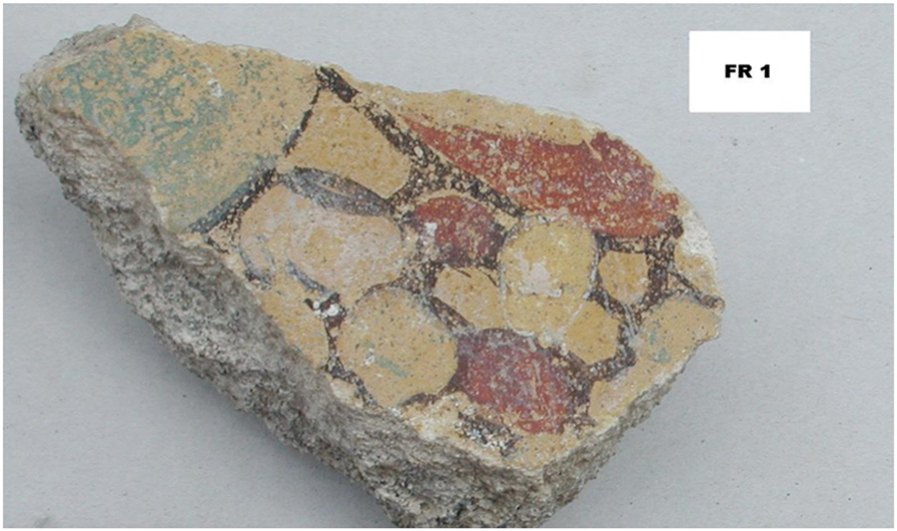
**A paint fragment of the excavation from *viridarium *(room 33), from where sample FR1 was collected.**

Four samples(samples FR1 (Figure 2), FR2, FR3 and FR4), from room 33 (*nymphaeum*), were taken from a few pictorial fragments discovered during an excavation in June 2004. 

The first sampling corresponded with the first phase of the research, which aimed at verifying the presence of organic binding media in the paintings to find out which paint technique used. To this end, nineteen samples were taken from rooms 2, 8, 9, 33, 41 of the *domus,* and from areas 6 and 11 of the Casa del Maiale (IX 9, c).

The second sampling corresponded to the second objective of the research: the identification of the organic substances used in the restoration. Eighteen samples were taken from areas 8, 41, 42, 43, 46, 47. The samples, taken from areas 41, 42 and 43, come from pictorial zones showing signs of flaking which indicates a loss of adhesive power. From areas 8, 46, 47, the samples were taken from zones where the pictorial pellicle was slightly bulging compared with surrounding zones.

**Figure 3:**
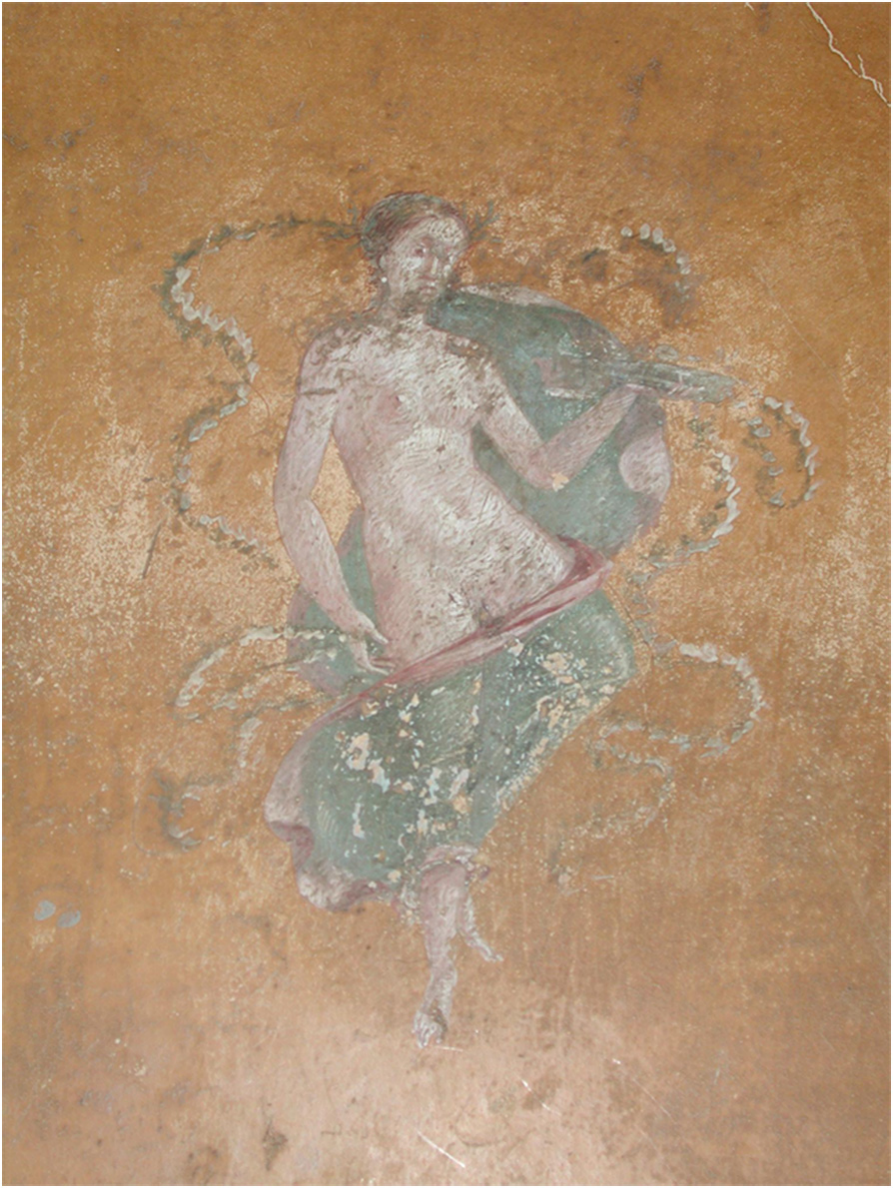
**Picture of Season in room 42 (the antechamber of the so-called erotic *cubiculum*, south wall, area 2 , pars mediana).**

**Figure 4:**
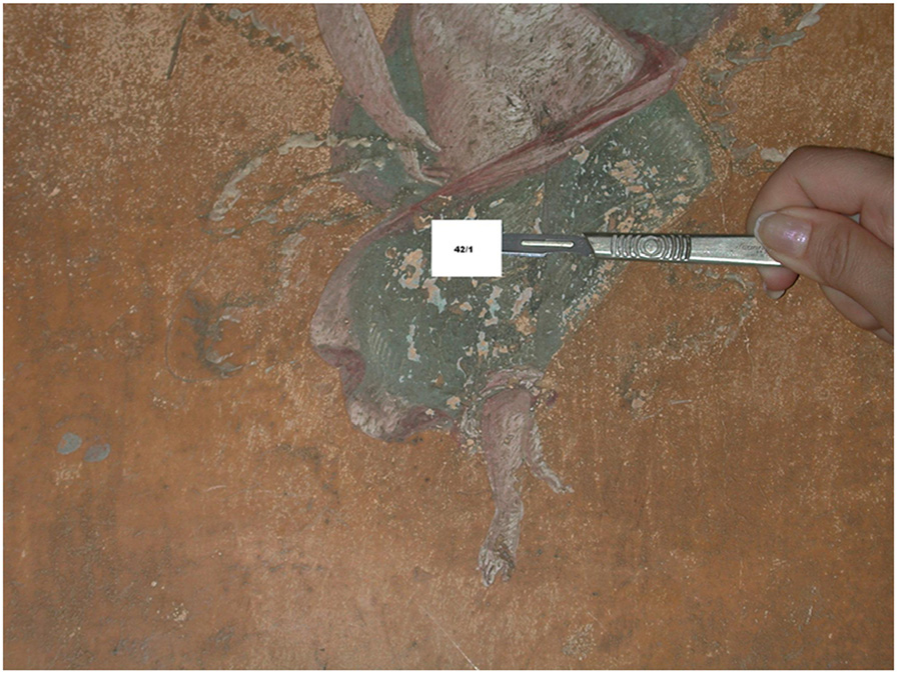
Sample 42/1 collected from the paint layer of the cloak of picture of Season.

**Figure 5:**
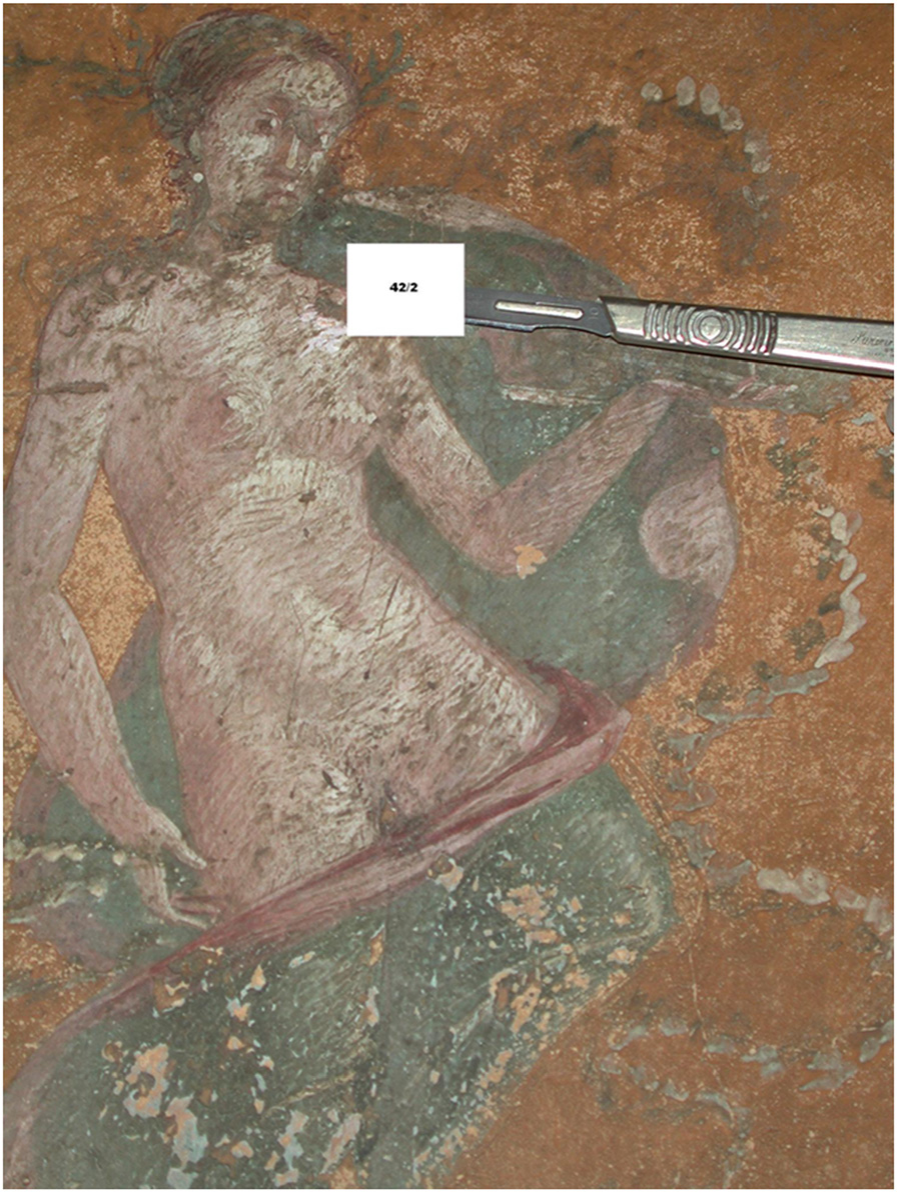
Sample 42/2 collected from the paint layer of the left shoulder of picture of Season.

Figure 3 shows the picture of Season in room 42 (the antechamber of the so-called erotic *cubiculum*), south wall, area 2 (pars mediana), from where sample 42/1 (Figure 4) and sample 42/2 (Figure 5) were collected. 

### Fourier Transform Infrared Spectroscopy (FT-IR)

The infrared spectroscopy investigation was carried out in attenuated total reflectance (ATR) employing a “Continuμm”- Nexus line micro spectrophotometer from ThermoNicolet equipped with a ZnSe crystal. Infrared spectra were recorded in the spectral range of 4000 to 650 cm^-1^, resolution 4 cm^-1^ and 32 scans.

### Gas Chromatography - Mass Spectrometry (GC/MS)

The analytical method used was based on a combined procedure for the characterisation of drying oils [11] and proteinaceous materials [8] on the same sample.

10 μg of heptadecanoic acid (10 μl of a 1 mg/ml solution), 10 μg of norleucine (10 μl of a 1 g/l solution), and 1 μg of norvaline (10 μl of a 0.1 mg/ml solution) per 1 mg of paint samples were added.

*Fatty acid analytical procedure* - The material was treated with 4 N-HCl in methanol (1 ml) and n-hexane (1 ml) for 2 h at 50°C. The n-hexane phase, which contains fatty acid methyl-esters, was used for gas chromatographic analysis (1 μl). For the analysis of the fatty acid derivatives, the GC oven temperature program was: 80°C for 2 minutes, then increased to 270°C at 20°C/minute, followed by a 6 minutes isothermal period.

*Amino acid analytical procedure* - After evaporation to dryness of the methanol phase, the residues were dissolved in 6 N hydrochloric acid (2 ml) and hydrolysed in a screw-capped container for five hours at 100°C in an oil bath, under nitrogen atmosphere. After evaporation to dryness, the hydrolysed residues were esterified using 3 ml of 2 N HCl in propan-2-ol at 90°C for one hour. After cooling, the solvent was evaporated under vacuum and the residue of the paint was dissolved in 0.2 ml of dichloromethane and derivatised with 0.2 ml of trifluoroacetic anhydride at 60°C during one hour. After cooling, the solvent was evaporated under vacuum and the residue of the paint sample was dissolved in 0.2 ml of dichloromethane, then the solution was used for gas-chromatographic analysis (1 μl). For the analysis of the amino acid derivatives, the GC oven temperature program was: 60°C for 3 min; 25°C/min to 260°C; then isothermal for 6 min.

A 6890 N Network GC System coupled to a to a 5973 Network Mass Selective Detector (Agilent Technologies) was employed. VF-5 fused-silica capillary column (30 m x 0.25 mm x 1 μm) coated with a 0.25 μm film of Methyl silicone (5% Phenyl), FactorFour, Varian Inc. (USA), was used for the separation, operated with temperature programming from 50°C (held for 3 min) at 25°C/min to 260°C (held for 2 min). The injector was kept at 280°C, while helium gas flow was approximately 0.66 ml/min. The splitless injector was set to 280°C with a 30 seconds purge off time. Mass-spectrometer conditions were as follows: interface temperature 280°C, ion source temperature 190°C, electron impact at 70 eV. The mass-spectrometer was operated in the selected ion monitoring mode (SIM). The following target ions for amino acid analysis were selected: m/z 140 for alanine (Ala), m/z 168 for norvaline (Nval), m/z 182 for leucine (Leu) and norleucine (Nleu), m/z 126 for glycine (Gly), m/z 166 for proline (Pro), m/z 164 for hydroxyproline (Hyp), m/z 184 for aspartic acid (Asp), m/z 198 for glutamic acid (Glu), m/z 91 for phenylalanine (Phe). Calibration of the instrument was done for each series of experiments, using a standard solution of amino acid and fatty acid derivatives. Internal standards were used and their response factors were calculated and applied to compensate for differences in detector response.

### Protein identification

The identification of the proteinaceous material in unknown samples can be performed by principal component analysis (PCA) of amino acidic percentage content data, using a reference data set of 101 reference samples containing egg, casein, and animal glue belonging to the paint reference collection of the Opificio delle Pietre Dure, Florence [12].

In particular, the PCA is performed, using PASW Statistics 18, on the correlation matrix of the data. The first two components account for 89,3% of the variance of the data. The database used for the clustering considers the following eight amino acids: alanine (Ala), glycine (Gly), leucine (Leu), proline (Pro), hydroxyproline (Hyp), aspartic acid (Asp), glutamic acid (Glue), and phenylalanine (Phe).

## Results and discussion

The paint samples have been analysed in two stages. At the beginning the study was aimed to research the proteinaceous and lipidic substances present in ther wall paintings of *Insula* del Centenario, using gas chromatography/mass spectrometry analysis. The chromatographic profiles revealed that none of the analysed samples contained amino acids or fatty acids, which indicates an absence of proteininaceous and lipidic materials. Clearly, these findings demonstrate that the wall paintings at *Insula* del Centenario and those in the Casa del Maiale were realized without these materials.

The analyses effected during the second phase were aimed at individuating the organic substances used during the restoration carried out in the nineteenth century. The twenty two samples collected were first examined under an optical microscope, then analysed with Fourier Transform Infrared Spectroscopy and finally with gas chromatography/mass spectrometry.

**Figure 6:**
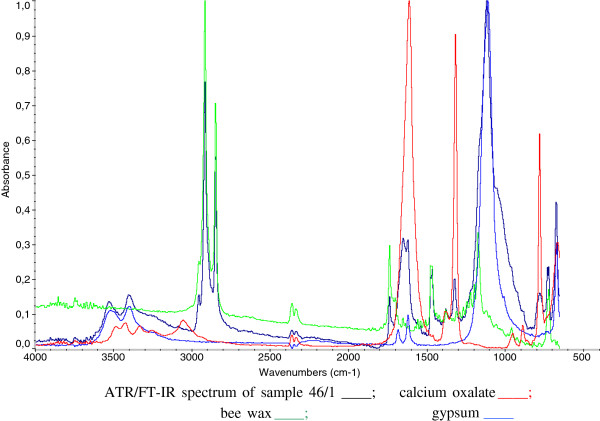
ATR/FT-IR spectrum of sample 46/1 ____; calcium oxalate ____; beeswax ____; gypsum ____.

**Figure 7:**
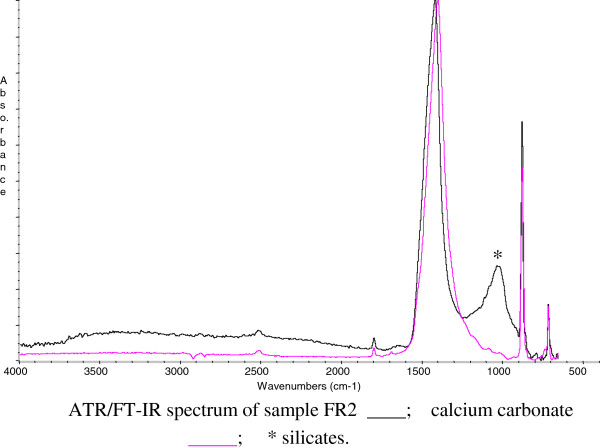
ATR/FT-IR spectrum of sample FR2 (discovered during the excavation on 2004) ____; calcium carbonate _____; * silicates.

The FT-IR analyses showed the presence of inorganic and organic compounds. Figures 6 and 7 show the ATR/FT-IR spectra of samples 46/1 and FR2, respectively. 

Concerning inorganic compounds, calcium carbonate on the wall paintings made on plaster was obvious; there was calcium sulphate dehydrate (gypsum) caused by the effect of sulphuric acid present in the atmosphere, this could interact with calcium carbonate in the plaster and transforms it into gypsum. Moreover, calcium oxalate was detected in the samples. This could be formed by the oxidation of organic materials.

Some samples showed the presence of organic material as evidenced by FT-IR spectroscopy by the bands at about 3000 cm^-1^, characteristic of C-H aliphatic stretching vibration, the band in region of 1700 cm^-1^ which corresponds to a carbonyl (C = O) stretching vibration and twin bands at 720 cm^-1^and at 730 cm^-1^.

**Figure 8:**
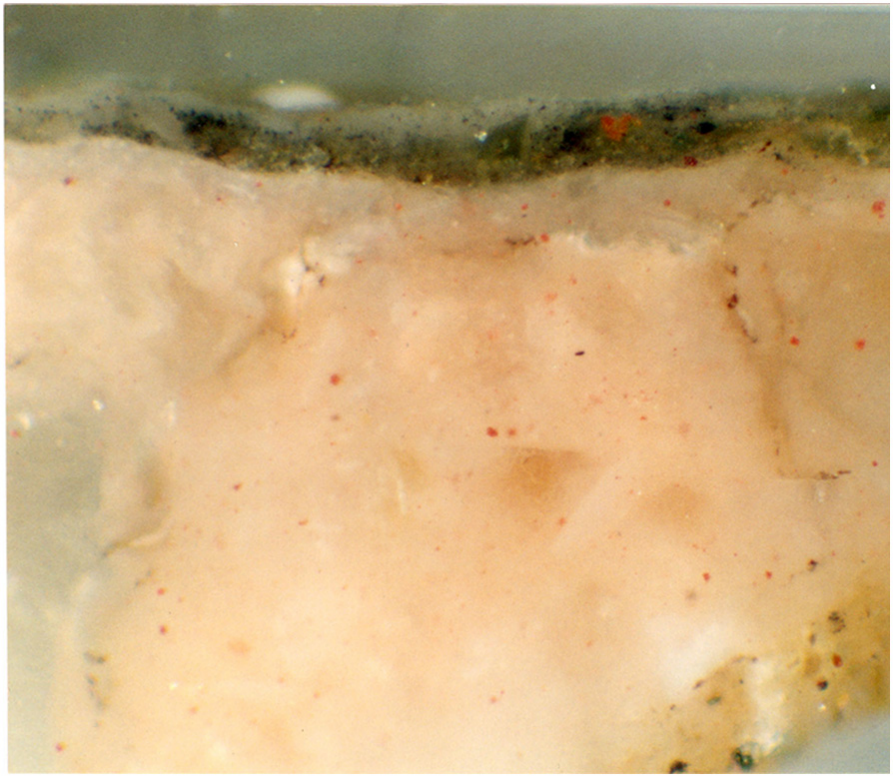
The cross section of the sample 42/3.

Through the interpretation of the spectra it has been possible to individuate the organic material as wax. Beeswax has been found in samples coming from the areas 41, 42, 43 and 46. Beeswax was not detected in samples coming from room 8, and in the sample from area 47. The presence of beeswax does not mean that the painting was realised with the *encausto* technique, because observation of the cross sections from the samples has shown that wax is only found on the surface of the paint, not into the pictorial layer, as one would expect from a real and proper binder. Figure 8 shows the cross section of the sample 42/3. 

Concerning samples coming from excavation material, investigations have revealed the presence of calcium carbonate and silicates, but they have also revealed the absence of gypsum, calcium oxalate and organic material. The use of beeswax for restoration works has been amply testified in Pompei through writings for having similarities observed in past restorations [5]; its usage was already recommended in antiquity for the protection of wall paintings [13].

For the GC/MS investigations, the derivatization procedure selected consisted of two main steps: the first one devoted to the lipidic components, the second to the proteinaceous material. Two chromatograms were collected for each sample: the first one from fatty acid derivatives, the second from amino acid derivatives.

**Figure 9:**
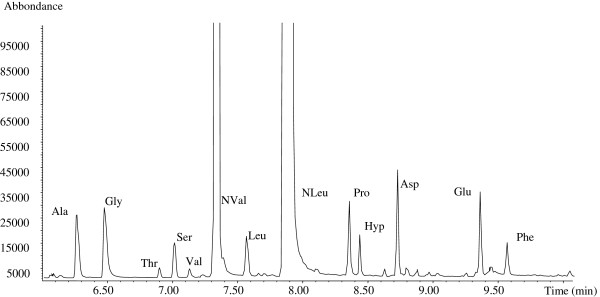
**GC/MS profile of sample 41/1. **Experimental conditions are reported in the text. Ala = alanine, Gly = glycine, Thr = threonine,Ser = serine, Val = valine, NVal = norvaline (internal standard), Leu = leucine, Nleu = Norleucine (internal standard), Pro = proline, Hyp = hydroxyproline, Asp = aspartic acid, Glu = glutamic acid, Phe = phenylalanine.

**Figure 10:**
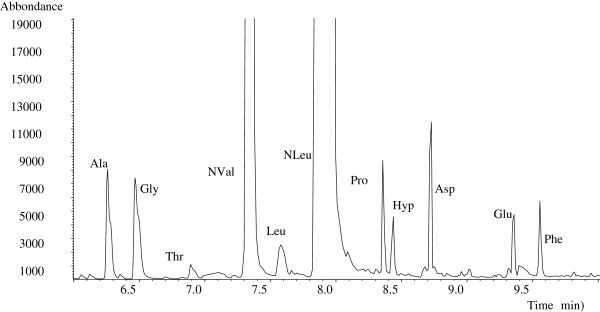
**GC/MS profile of sample 43/1. **Experimental conditions are reported in the text. Ala = alanine, Gly = glycine, Thr = threonine, NVal = norvaline (internal standard), Leu = leucine, Nleu = Norleucine (internal standard), Pro = proline, Hyp = hydroxyproline, Asp = aspartic acid, Glu = glutamic acid, Phe = phenylalanine.

The chromatographic profiles of the samples collected in areas 41, 42 and 43 showed the presence of amino acids, and the absence of fatty acids owing to drying oils. The Figures 9 and 10 show the chromatographic profiles obtained from samples 41/1 and 43/1, exemplifying rooms 41 and 43. 

The chromatographic investigations carried out on samples collected from the walls of rooms 8, 46 and 47, and those collected from excavation fragments have not shown any evidence of proteinaceous or lipidic materials.

Table 1 reports the percentage content of amino acids found in the ancient samples. In order to verify the classification of the proteinaceous binder present in the sample, we applied PCA analysis to amino acid percentages, comparing the data obtained by this procedure with data obtained previously [14,15], and relative to egg, animal glue and milk. 

**Table 1:** Relative peak areas, corrected using response factors (normalized 100%), of amino acid derivatives of the samples collected on June 2004

**SAMPLE**	**Ala**	**Gly**	**Leu**	**Pro**	**Hyp**	**Asp**	**Glu**	**Phe**
**41/1**	15,6	20,6	8,6	8,4	3,8	20,6	15,1	7,3
**41/2**	15,9	17,5	9,5	8,7	2,0	21,1	20,1	5,2
**41/3**	14,7	22,1	8,8	9,6	3,5	17,6	16,3	7,4
**42/1**	6,6	16,4	0,0	11,1	0,0	22,8	25,0	18,1
**42/2**	12,3	18,7	9,2	6,7	2,0	24,5	19,6	7,0
**42/3**	12,3	18,0	9,1	6,9	1,9	23,3	19,2	9,3
**42/4**	18,0	12,4	8,4	9,1	0,0	25,7	21,6	4,8
**42/5**	17,2	19,1	10,0	10,1	1,6	14,5	21,6	5,9
**42/6**	8,0	11,2	9,8	10,5	0,5	27,7	27,3	5,0
**42/8**	19,0	19,7	8,5	6,4	4,1	22,9	13,0	6,4
**43/1**	18,5	23,0	9,6	6,3	4,9	21,9	8,9	6,9
**43/2**	15,9	19,7	9,1	7,0	1,7	20,6	18,7	7,3
**43/3**	18,0	25,5	10,6	5,6	1,9	17,2	14,0	7,2
**43/4**	18,9	29,8	6,6	7,5	1,9	13,3	16,4	5,6

**Figure 11:**
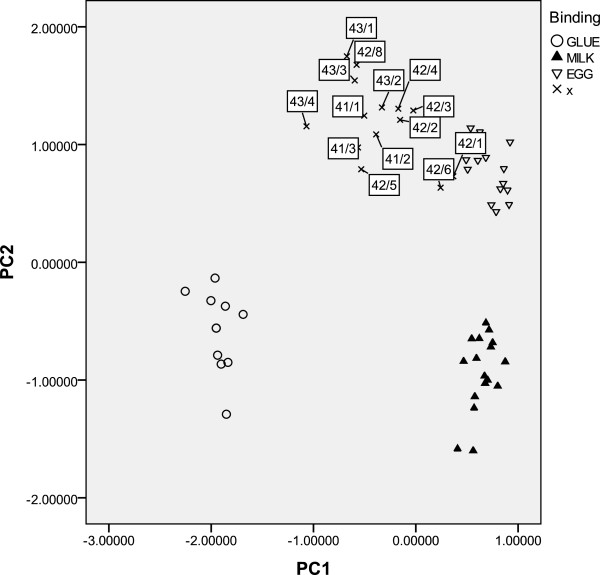
**PCA score plot of amino acid percentage data of reference samples containing egg , animal glue and milk binders and of the paint samples. **The relative **amino** acid percentage content of the Pompei samples is reported in Table 1.

The PCA plot (Figure 11) shows that the samples are grouped near to the egg cluster. The main limitation of an analytical approach to proteinaceous paint media identification based on amino acid quantitative determination is the presence of mixtures of different proteins in the same sample. Due to the position of the samples near to egg cluster and to our osbervation in the chromatogram of some samples of low signal of hydroxyproline, the hypothesis of the occurrence of egg together to animal glue, as minor component, seems reasonable. The hydroxyproline is the marker of the animal glue. 

Table 2 shows the results obtained studying the twenty two paint fragments sampled on June 2004. 

**Table 2:** Analytical results of the paint fragments sampled on June 2004

**SAMPLE**	** FTIR SPECTROSCOPY ANALYSES**	** GC/MS ANALYSES**
**41/1**	Beeswax, calcium carbonate	Egg, animal glue
**41/2**	Beeswax, calcium carbonate	Egg, animal glue (trace)
**41/3**	Beeswax, gypsum, calcium carbonate	Egg, animal glue
**42/1**	Beeswax, gypsum, calcium oxalate	Egg
**42/2**	Beeswax, calcium carbonate, calcium oxalate	Egg, animal glue
**42/3**	Beeswax, proteinaceous materials	Egg, animal glue (trace)
**42/4**	Gypsum, proteinaceous materials	Egg
**42/5**	Beeswax, gypsum, calcium carbonate	Egg, animal glue (trace)
**42/6**	Gypsum	Egg
**42/8**	Gypsum	Egg, animal glue
**43/1**	Beeswax, gypsum, calcium oxalate	Egg, animal glue
**43/2**	Gypsum, calcium carbonate	Egg, animal glue (trace)
**43/3**	Gypsum, calcium carbonate, silicates	Egg, animal glue (trace)
**43/4**	Beeswax, gypsum, silicates	Egg, animal glue (trace)
**8/1**	Gypsum, calcium oxalate	----
**8/2**	Calcium carbonate, gypsum	----
**46/1**	Beeswax, gypsum, calcium oxalate	----
**47/1**	Calcium carbonate, silicates	----
**FR1**	Calcium carbonate, silicates	----
**FR2**	Calcium carbonate, silicates	----
**FR3**	Calcium carbonate, silicates	----
**FR4**	Calcium carbonate, silicates	----

## Conclusions

The results of the first phase of the research have shown that no organic substance has been used in the nineteen samples studied, excluding their usage. We reinforce the hypothesis of the fresco technique. The hypothesis that the technique used by the painters working at *Insula* del Centenario (and those working in the Casa del Maiale) was in fresco fits in with the larger context of painting in Roman times, a technique now individuated, with certainty, as fresco painting. The data that have emerged from this first phase match convincingly those to be found in recent literature on the subject [2-5].

The results of the second phase of the research revealed the presence of organic materials. The presence of beeswax, that emerged from the analysis by means of spectroscopy FT-IR, can be explained as residue of past maintenance works carried out to restore the original colours of the paintings. The use of this product for restoration works has been amply testified in Pompei through chronicles as having similarities observed in past restorations; its usage was already recommended in antiquity for the protection of wall paintings [5]. By observation of the cross sections of the samples, it possible to localise the wax on the pictorial surface. It may be presumed, therefore, that the beeswax was not used as a binder and may not have been part of the wall painting procedure. The absence of wax, noted in the samples collected from paint material taken from the specimen of the fragments of the excavation from viridarium confirms the validity of such an hypothesis.

The research through the investigations GC/MS has emerged the presence of proteinaceous material in the rooms of the private apartment (41, 42, 43). In particular, egg is present in the room 42, and egg with animal glue, as minor component, in the samples collected in the rooms 41, 42, 43. It was noted that these samples came from the zones where the pictorial surface was flaking. The egg with animal glue could have been the cause of the flaking of the surface brought about by the loss adhesive power of these substances. Its presence ties up with past restoration interventions aimed at restoring the visual unity of the painted surface. It is, therefore, a question of binding substances used for touching up or repaint the incomplete parts of the pictures. Obviously, the samples collected from fragments on the excavation site have not shown the presence of this type of substances.

What is striking from an archealogical point of view is the individuating of these organic materials in rooms 41, 42, 43 only. They are certainly remarkable for the quality of the preserved decoration, but the *oeucus* 8 (its importance is indicated by being placed at the centre of the domus), where these materials are not present, is equally or even more remarkable. They are not to be found in the large picture of the *nymphaeum* either, though it is one of the “jewels” of the house.

It is probable that the difference in preservation treatment is due to a particular and precise phase in the history of Pompeyan painting preservation, during which attention was exclusively directed to the “figurative” parts, that is, to painted pictures inserted in the decoration. This attention led to effective interventions which nevertheless were too invasive, such as the excision of the pictures of the room 41 and their positioning on a lead support to isolate them from the humidity of the walls. However, no particular attention was paid the whole decoration of the salon, which was allowed to get lost though it was one of the best examples of the style III of all Pompey. The covering of the salon has in fact been placed in the 20^th^ century (in the sixties). The problem relative to the Pompeyan pictorial restoration is that the archives very rarely contain precise information as to the modality of the restoration interventions, or the materials used, and it is only by associating the archive research with the archeometric analyses that it was possible to reconstruct a history of the Pompeyan pictorial restoration; an indispensable introduction to new, more conscious and effective preservation interventions.

## Competing interests

The authors declare that they have no competing interests.

## Authors’ contributions

AC conceived and supervised this research work, carried out the analyses, the validation studies, and drafted the manuscript. SS contributed with her archaeological knowledge and archaeometric assessment. Both authors read and approved the final manuscript.
